# Truth and consequences

**DOI:** 10.1111/meta.12644

**Published:** 2023-07-03

**Authors:** Polly Mitchell, Alan Cribb, Vikki Entwistle

**Affiliations:** ^1^ Centre for Public Policy Research King's College London United Kingdom; ^2^ Health Services Research Unit and Department of Philosophy University of Aberdeen United Kingdom

**Keywords:** applied philosophy, methodology, philosophical methods, public policy

## Abstract

In his 1987 paper “Truth or Consequences,” Dan Brock describes a deep conflict between the goals and virtues of philosophical scholarship and public policymaking: whereas the former is concerned with the search for truth, the latter must primarily be concerned with promoting good consequences. When philosophers are engaged in policymaking, he argues, they must shift their primary goal from truth to consequences—but this has both moral and methodological costs. Brock’s argument exemplifies a pessimistic, but not uncommon, view of the possible shape and nature of applied philosophy. The present paper paints a richer and more optimistic picture. It argues that the difference between theoretical philosophy and applied philosophy is not best understood as a choice between truth and consequences. On the contrary, applied philosophers engage in forms of truth‐seeking that are properly concerned with consequences—including the consequences of philosophical practice itself.

## 1 INTRODUCTION

In his 1987 paper “Truth or Consequences,” Dan Brock candidly describes his experience as a staff philosopher with the President's Commission for the Study of Ethical Problems in Medicine—a congressionally mandated group in the United States, which operated between 1978 and 1983.^1^ Reflecting on his experiences, Brock asserts that there is a deep conflict between the goals and virtues of philosophical scholarship and public policymaking: whereas the former is concerned with the search for truth, notwithstanding the social consequences thereof, the latter must be primarily concerned with promoting good consequences. He argues that when philosophers engage in policymaking, they must shift their primary goal from truth to consequences, and that a failure to do so is irresponsible. He warns, however, that such a shift has both moral and methodological costs.

In this paper, we argue that Brock is right to highlight—as have others—the potential tensions between scholarly philosophy and policymaking, but the conclusion that these tensions amount to a “deep conflict” reflects a needlessly pessimistic view of applied philosophy. We offer an alternative to a “truth or consequences” dichotomy, seeking to paint a richer and more optimistic picture of applied philosophy.

Applied philosophy, we argue, is not a morally and methodologically compromised form of philosophy but a way of doing philosophy that is distinctively responsive to (non‐philosophical) practice in its goals, scope, content, and ways of working. Applied philosophers can play an “insider‐outsider” role vis‐à‐vis the institutions and practices that they engage with—recognising their goals and conventions while not being bound by them.

The difference between scholarly and applied philosophy is not, we think, best understood to consist in a respective concern with “truth” and “consequences.” On the contrary, applied philosophers engage in truth‐seeking that is, to some extent, properly concerned with consequences—including the consequences of philosophical practice itself. Philosophers who engage in the messy reality of public and professional decision‐making do not thereby dilute or distort their philosophical practice. Rather, their engagement can expand and enrich their philosophy, creating new opportunities and spaces for philosophical thinking.

## 2 TRUTH OR CONSEQUENCES

We begin by summarising Brock's account of the “deep conflict” (Brock 1987). We link Brock's account to similar arguments made elsewhere and suggest that these arguments have serious implications for the prospects of applied philosophy more broadly.

Alongside other staff serving the President's Commission—including philosophers and non‐philosophers—Brock advised elected commissioners on the content of reports on bioethical issues, which made policy recommendations. His reflections on his tenure with the commission begin with a statement of the respective aims of scholarly philosophy and public policy. Philosophy, he argues, involves a search for the truth regardless of the social consequences of so doing. For the academic philosopher, nothing is immune from criticism; all assumptions, claims, beliefs, and arguments must stand ready to be challenged and defended. And while philosophers do not always meet this standard, unconstrained truth‐seeking is a central philosophical virtue. Making public policy, on the other hand, requires careful attention to the consequences—for policy and ultimately for the people affected by policies—of statements, decisions, and actions in the policymaking process.

To illustrate this divergence, Brock describes working on a report about withdrawing life‐sustaining treatment. He recounts that many of the commissioners believed that killing someone is far more seriously wrong than allowing him to die. They also believed that withdrawing life support amounts to allowing a patient to die of his disease, rather than killing him. While Brock agreed with the commissioners that it is sometimes morally permissible to withdraw life‐sustaining treatment, he thought the commissioners' reasons for believing this were unsound. He recognised, however, that if he were able to convince the commissioners that there is no morally relevant distinction here, this might lead them to believe that it is *not* morally acceptable to withdraw life‐sustaining treatment, and to make policy recommendations accordingly. Such an outcome could, in Brock's eyes, produce worse policy and worse consequences for the public. His worry, then, is that pursuing philosophical truth by challenging the soundness of the commissioners' arguments could harm real people.

To avoid this outcome, commission staff had to tailor what they said and did with an eye to the consequences of their actions. As well as allowing bad arguments to slide, this might involve refraining from raising controversial topics or extreme views, in order not to lose credibility or risk losing the opportunity to speak, or the likelihood of being listened to, in the future. They had to “sell” policy proposals to their colleagues, and this necessitated “packaging” them in ways that appealed to individuals with decision‐making power.

Although Brock takes it to be morally responsible for philosophers working in policy contexts to adopt the aims of policymakers, setting aside their scholarly philosophical aims, he thinks this carries costs. In part these costs are methodological in nature: it is a methodological virtue of philosophy that it engages in an unconstrained and ecumenical search for truth. Giving weight to the consequences of one's statements and actions can require philosophers to play “fast and loose with the truth . . . in a way that is inimical to the scholarly academic enterprise” (1987, 789). But Brock is also concerned about the moral costs of adopting policymaking aims. He suggests that to influence policy philosophers must sometimes intentionally strategise the arguments that they make publicly and the positions that they adopt and defend in order to promote optimal social consequences, rather than solely defending positions that they believe to be sound. Such strategising involves taking “manipulative attitudes towards others” (1987, 789). Brock concludes: “I believe the scholarly‐policy conflicts that I have cited here do give reason for thinking that philosophers' forays into the world of policy should best be limited and temporary, not full time and permanent. The philosophical virtues that enable philosophers to make effective, valuable, and distinctive contributions to the policy process are probably best maintained if their primary base and commitment remain in academic philosophy” (1987, 791).

While Brock's paper is a strikingly forthright expression of the idea that philosophy is best kept apart from the complexities of real‐world decision‐making, this view is echoed elsewhere. It is signalled, for example, by Alberto Giubilini and Francesca Minerva, in their open letter written in response to—and expressing surprise about—widespread critique of their paper defending the permissibility of “after‐birth abortion” (Giubilini and Minerva 2013): “[W]e never meant to suggest that after‐birth abortion should become legal. . . . Laws are not just about rational ethical arguments, because there are many practical, emotional, social aspects that are relevant in policy making (such as respecting the plurality of ethical views, people's emotional reactions etc). But we are not policy makers, we are philosophers, and we deal with concepts, not with legal policy” (Giubilini and Minerva 2012).

The idea that there are “practical, emotional, social aspects” of policymaking that sit outside the concerns of philosophy circumscribes the scope of philosophy fairly radically; here “rational ethical argument” seems to be reserved for a “cool hour,” away from the heat and noise of practical decision‐making (Williams 1982). As well as describing their intention to execute “a pure exercise of logic,” Giubilini and Minerva say that their “article was supposed to be read by other fellow bioethicists who were already familiar with this topic and our arguments” (Giubilini and Minerva 2012), thereby emphasising the presumed separation of both philosophers and philosophising from other social domains. In his excellent essay on philosophical advice, David Archard differentiates the attitude expressed by Giubilini and Minerva—“I didn't mean to advise”—from Brock's worry about the moral and methodological costs of advising policymakers badly in an attempt to second‐guess and avert misrepresentation, misunderstanding, and misuse of arguments (Archard 2021). But both seek to distance “philosophy proper” from the emotions, psychological states, practical rationality, and socio‐political context of decision‐makers, seeing engagement with and responsiveness to these as a departure from good philosophical methods and practice.

We have identified a perceived conflict between philosophers' scholarly aims and their aims when engaged in policymaking. But if such a conflict exists, it potentially has far‐reaching implications. There is potential for conflict between the professed scholarly philosophical virtues and the goals and aims of many non‐academic practices. Applied philosophers whose guidance feeds into decision‐making in health care settings, for example, may similarly need to consider the effects of their assertions and arguments on decision‐making outcomes, and intentionally strategise their philosophical practice to ensure good social consequences. So too may those who seek to influence practice in education, legal institutions, commerce, the military, technological development, and any other areas of practice that could affect, for better or for worse, the lives and life chances of many individuals.

Claims of a schism between philosophy and practice have weighty implications for philosophers who want to influence practice in these and other social domains. For they suggest that, at least in so far as it involves strategic concern with social consequences, this work ought to form only an occasional part of their philosophical practice at best. This sounds like bad news for the many applied philosophers whose work engages with non‐scholarly practice in a sustained way—often involving ongoing collaboration with professionals, and emphasis on practice‐oriented problems and questions.

## 3 THE MORAL COSTS OF POLICYMAKING

In this section we set aside the supposed methodological costs of applied philosophy and focus on the moral costs. We argue that the idea of a “deep conflict” between scholarly philosophy and policymaking arises from, and makes sense only in relation to, unusual examples of high‐pressure and high‐stakes decision‐making, where tensions between scholarly virtues and a concern about foreseeable and damaging public policy consequences come to a head. There *are* dilemmatic moments in public policymaking where tensions are profound, but this need not imply that there is some permanent chasm separating philosophy from policymaking. The idea that the conflict is “deep” in this sense, we suggest, places too much weight upon a narrow and outcomes‐oriented view of the aims of policymaking and the contributions of philosophers. By contrast, we stress that, for the most part, manipulative attitudes are an obstacle to good policymaking and that policymaking processes that incentivise manipulation are—in this respect, at least—*bad* policymaking processes. Policymakers, and philosophers who support them, need to engage with and consider their impact on policymaking processes as well as policy outcomes.

Frances Kamm (1990) highlights aspects of the role of philosophers working on public commissions and in public advisory roles that fall outside Brock's discussion. Kamm argues that philosophers serve primarily as educators to commissions—and, by extension, other such institutions—and that, in this role, their duties to the commission and its members can take precedence over their duties to the public. A philosopher serving on bioethics commissions may have a duty to speak the truth to commissioners, including identifying implications of and problems with commissioners' views; telling the commission her own judgement about what the bottom line should be and her reasons for thinking this to be so; and sharing other reasonable philosophical views and arguments that she does not herself hold. In so doing, she can guide colleagues in genuine philosophical reflection. Duties to promote the public good or to uphold philosophical virtues may be secondary to this, meaning that the philosopher may, in principle, be morally required to fulfil her educational role even if this will lead to worse social consequences. Kamm emphasises the role of the philosopher in scrutinising and supporting the *process* of policymaking and not just in securing good *outcomes*.

But even where outcomes are concerned, bioethics commissions plausibly have more complex aims than merely recommending, and helping to bring about, policies with good social consequences. They might, for instance, also play an important role educating the public and informing public debate—setting the public bioethical agenda; clarifying concepts; and developing factual, conceptual, and moral nuance to better understand what is at stake (Dzur and Levin 2004). Such aims might be only indirectly related to policymaking. It could take time and much further discussion before thoughtful policies result from the guidance and recommendations of bioethics commissions. But such guidance can nonetheless play a role in setting the direction and tone of public debate and in ensuring that it is factually and morally sophisticated. Institutions like bioethics commissions are not merely a means to producing better policies, they are also part of a policymaking process and a landscape of public reason that seeks to embody, promote, and produce all sorts of goods other than policy outcomes.

Brock asserts that “the first concern of those responsible for public policy is, and ought to be, the consequences of their actions for public policy and the persons that those policies affect” (1987, 787). This doesn't, of course, rule out policymakers—and policymaking philosophers—having other aims as well, such as those mentioned in the preceding paragraphs. But it's implausible, we think, that the “first concern” of policymakers that Brock identifies is lexically prior to any other aims that they might have; there are some immoral policymaking processes that are clearly unjustified, regardless of any beneficial policies they might generate. Process‐related concerns will at least sometimes trump outcome‐related concerns. It is therefore not necessary to commit to Kamm's strong claim that a philosopher's duties to promote good social consequences are secondary to her duties to the commission to recognise that the diverse commitments of policymakers are sometimes in tension with one another and it might not be possible to pursue them all at once.

One way forward here would be to broaden our conception of the “social consequences” of policymaking, such that the set of public policy consequences that policymakers ought to take into account also includes the bioethical education of the public, the quality of public reasoning and debate, and so on. The “good social consequences” that Brock identifies as the first concern of policymakers, seen through this slightly broader frame, might well also include consideration of the decision‐making process itself—the values it embodies, the goods it produces, and the ways that it contributes to public life. On this broader view, a moral philosopher's role in a commission or policy‐guiding panel need not—indeed, should not—merely be a matter of ensuring that the commission recommends policies with good consequences. They also have a responsibility to engage with, and treat as an object of philosophical inquiry, the operation of the commission and its moral implications.

This suggests that manipulative attitudes shouldn't be understood to be an unfortunate cost of doing philosophy in policymaking contexts. Rather, in so far as they feature in or are incentivised by the decision‐making process, this should itself be a site of engagement for the policymaking philosopher. Manipulative attitudes are, in theory, compatible with the aim of bringing about good social consequences in the narrower, more instrumental sense—certain good outcomes can be reached by morally unsettling means. But it's less clear that they are compatible with bringing about good social consequences in the broader sense, in so far as manipulation, and the lack of transparency that it involves, is incompatible with some of the central virtues of good public policymaking. If we frame the aims of public policymaking too narrowly, such manipulation appears as an unfortunate side effect of pursuing such aims, rather than a potential obstacle to their pursuit and fulfilment. This is not to suggest that manipulation can never be justified in the course of policymaking but rather to suggest that a policymaking process that systematically incentivises it in order to recommend or generate policies can be thought to be, to that extent, a *bad* policymaking process. And it is thus a legitimate target of critique and debate for philosophers supporting policymaking work, rather than a reason for them to limit their policy engagement.

Public policymaking may occasionally involve high‐stakes and high‐pressure dilemmas. While philosophers can seek to shape policy processes, they may not always be in a position to do so early enough, or their advice or example may not be heeded. In exceptional cases the result may be that they judge they are in a position, and have an obligation, to avert what they see as dangerous policymaking, perhaps even at some expense to their professional integrity. This, however, is best seen as illuminating the boundaries of philosophy in these contexts rather than as something characteristic of it.

The fact that Brock writes about his experiences on the President's Commission and the associated moral tensions suggests that he does recognise this as a site for philosophical and moral engagement. He makes this critique from the outside, however, as part of a metaphilosophical discussion about the appropriate role and conduct of philosophers. But such engagement with the policymaking process can and sometimes should occur *within* the process, as part of a philosopher's role. The dilemmas Brock discusses are characterised as one‐off, high‐pressure decisions, but we have suggested that policymaking philosophers need to take a broader and longer‐term view of policymaking processes. The role of the philosopher in policy environments is not just to help bring about good social consequences but more broadly to engage with the reality of the practices in which she is involved. These practices should be understood not as mere means to ends about which moral and conceptual assessments can be made but also as ends in themselves, worthy of philosophical scrutiny as part of a philosopher's public role.

## 4 INSIDER‐OUTSIDER ROLES

In the previous section, we argued that applied philosophers have opportunity, and perhaps responsibility, to critically reflect on the assumed conventions and goals of the practitioners and practices with which they engage. Here we develop these thoughts into a broader discussion about the scope and nature of applied philosophy. We argue that applied philosophers play an insider‐outsider role: they are responsive and attentive to the existing goals and norms of practice, while maintaining a critical, reflective attitude towards them that enables a more expansive and revisionary perspective.

Methodological work in applied ethics provides a good starting point for thinking about the nature of applied philosophy. The idea that applied ethics is not just a matter of applying normative theories to particular problems is well‐trodden ground (Beauchamp 2005; Wilson 2009; Wolff 2019). Arthur Caplan makes the point forcefully: “It is simply naive to think that a well‐trained philosopher can step boldly into the emergency room or neonatal unit and immediately dissolve moral conundrums by dint of expertise in moral theory” (1980, 27). Applied ethics tends to be concerned with standards, codes of practice, roles, and virtues that are internal to particular social practices and professional activities, as well as broader moral principles and conventions. Much of the content of applied ethics derives from observation and interpretation of practice—understanding what people actually do and think they are doing is taken to be absolutely central to understanding what they *should* do. The starting point for applied ethicists, on this account, is not neat and tidy moral rules, theories, and principles, which are then applied to complex and messy situations, but the messy and complex world itself, which gives rise to conceptual and theoretical objects (Walker 2007).

This need not imply a strong casuist or anti‐theoretical approach. There is much work in applied ethics that defends relatively fixed and determinate mid‐level and even high‐level moral principles and rules, and reflective equilibrium has been widely adopted as a method of moral reasoning and justification (Arras 2007; Beauchamp 1984; Beauchamp and Childress 2019; Daniels 1996). But it does suggest that applied ethics looks first to practice in identifying and characterising—though not exhaustively—relevant questions, issues, and concepts. This might comprise engagement not only with professionals but also with the field of practice more broadly, including observation of and dialogue with other people who are involved or implicated in it—patients, service users, customers, students, members of the public, and so on—and thus to incorporate diverse experiences of ethical concern (Farmer and Campos 2004). This sociological tendency need not be limited to localised areas of applied ethics. Indeed, a realistic understanding of human psychology and behaviour might be thought a necessary underpinning for broader ethical theorising (Flanagan 1991). Nor need this philosophical approach be limited to *ethical* analysis—applied philosophy more broadly can be understood to be grounded in the actual characteristics and behaviour of people and even objects. Theorising and conceptual analysis in the philosophy of science might be thought to require deep engagement with scientific practice (Cartwright 1999; Dupré 1993). And understanding ordinary language and medical practice is arguably central to philosophical work on the definition of health and disease (Broadbent 2019; Glackin 2019; Kitcher 1996).

Reflecting on an approach to philosophy and public policymaking that is rooted in engagement with practices, Jonathan Wolff (2011) argues that a key question for philosophers working with policymakers is what would be for the best when “starting from here”—that is, based on a thorough understanding of the prevailing circumstances, including resources and constraints, available practical, legal, and political mechanisms, and the kinds and levels of real‐world disagreement that have to be navigated. Rather than focus on the risks of “contamination” from immersion in the field, Wolff highlights the moral hazards of starting too far away from the field. The hazards here include irrelevance but also, when one is not ignored completely, significant risks of causing harm. Wolff is not dismissive about the contribution of ideal theory to applied philosophy. On the contrary, he sees it as playing a crucial role in helping to challenge prevailing assumptions and bringing about change in the long term: “[W]e can distinguish two roles for philosophical input into policy debate. At the sharp end of policy, when an issue is being discussed and a practical outcome is being sought, philosophers have to operate in a pragmatic mode. For if their recommendations do not respond to the values people actually hold, then they will be left out in the cold. But a longer‐term project is also possible, and arguably more valuable: to set out arguments and visions of other ways of doing things that might hope to shape the values that people hold” (Wolff 2019, 261).

This unpacking of longer‐term and nearer‐term facets of applied philosophy is, we suggest, a helpful counterweight to any notion of a sharp distinction between “philosophy proper” and practice. It suggests that scholarly virtues may, to some extent, take on different shapes and emphases depending upon the circumstances in which philosophers are working and their variable purposes. It also indicates that the ethical balancing acts in applied philosophy precede the high‐stakes dilemmas with which we began and, in particular, include decisions about how to balance or combine more immediate pragmatic contributions with longer‐term and more foundationally challenging contributions.

When philosophers take the realities of practice as a starting point, they take on some of the concerns of practitioners. But this does not mean that applied philosophers must adopt the ends of practitioners as their own ends and trade away the goals of philosophy. We suggest, instead, that applied philosophers play an insider‐outsider role in their relationship with non‐philosophical practice and practitioners: while the concerns of practitioners shape and constrain applied philosophical work, the concerns of practitioners and philosophers are certainly not identical. Attempts to critique and reframe existing practice are central to applied philosophy; in order to retain her distinctive role and approach, the applied philosopher must remain to some extent an outsider to the practice that she engages with and seeks to influence.

Applied philosophers typically evaluate the practices upon which they focus in order to critique, defend, or reframe them, and this evaluation might take the form of ethical, conceptual, or logical analysis (Beauchamp 1984). But to be meaningfully *applied*, philosophical work is constrained in its subject matter and its methods. The objects of concern, questions, arguments, and solutions that form the basis of applied philosophical work are limited by the scope—or at least the potential scope—of a given field of practice. This is not to say that philosophers can only repeat ideas that are already expressed by practitioners, or only engage with existing practice, but it does mean that applied philosophical work is constrained by its intelligibility, its usefulness, and its reasonability to practitioners and other stakeholders. If philosophical work does not have any implications for or potential impact on a field of practice, as understood by those who work in or are affected by it, then it is not clear that it is meaningfully *applied*. So, when applied philosophers engage in criticism and reframing of practices or make recommendations about how they should change and develop, they don't entirely occupy an outsider perspective—they keep one foot in the door.

But applied philosophical work differs from the work of practitioners and policymakers in important respects. Generating “good consequences”—that is, measurably changing outcomes for the better in some domain—is one way of conceiving how philosophers can change a field of practice. For example, philosophical engagement with patients and health care practitioners can lead to improvements in service outcomes when it highlights moral shortcomings or unjustified inconsistencies in current practice. But genuinely applied philosophy also has the potential to change health care practice in more fundamental and systemic ways. By encouraging reflection on the goals and vision of health care, challenging prevalent hegemonies, introducing conceptual and ethical nuance into discussion, and normalising dialogue and conversation about genuinely thorny, complex problems without the expectation of resolution, philosophers have the potential to change health care institutions and their processes. Such changes might well result in better outcomes for patients, and perhaps staff too, but also generate critical reflection on the nature of good health care and new frameworks for conceptualising success and failure that can underpin longer‐term transformation of practices. The adoption of the goals of practice by applied philosophers, then, is not entirely straightforward, as it makes space for the reconsideration and reconceptualization of those same goals in potentially radical and practice‐affecting ways.^2^

A familiar example of this phenomenon is the critiques of the biomedical model of medicine and the proposal of explicitly normative alternatives that highlight, for example, personal ambitions and goals (Nordenfelt 1995), capabilities (Venkatapuram 2011), narrative (Charon 2006), phenomenology (Carel 2016), and population health (Valles 2018). At its best, this kind of philosophical engagement has challenged the medical community to rethink *what* and *who* medicine is for, to reconsider the definition and extension of health, and to reframe how to think about success. Ethical and conceptual reflection that reframes health care practices and the nature of “good” health care in this way does not straightforwardly generate good consequences. Indeed, it may, in some sense, bring about *bad* social consequences. If “good” health care is reframed in such a way that reveals major deficiencies in current practice, philosophical inquiry has in effect “created” a whole new set of bad consequences, which might show up in qualitative and quantitative measures and in testimony. Our use of “created” here is slightly tongue in cheek, but not entirely; for the deficiencies may be literally unrecognisable as such under other value frameworks, and so be brought into existence, from an institutional perspective, by conceptual reframing. Of course, good consequences might well also result—but “good” according to the new conceptual frameworks, and not necessarily to the ones they replace.

The manner in which conceptual reframing represents a view from “outside” practice needs some qualification. For reconceptualisation of practice can highlight different perspectives *within* a field of practice, emphasising marginalised views or experiences and demonstrating the ways that dominant conceptual frameworks are less advantageous to or make less sense of the experience of some people. This suggests that the “outsider” perspective occupied by philosophers might sometimes be internal to a field of practice but external to some of its prevailing attitudes, institutions, and authorities. This includes attention to the perspectives of those on the receiving end of policy and practice interventions and those at the bottom of social hierarchies within those groups (Farmer and Campos 2004; Scully 2017; Walker 2007). More radical philosophical reflection can thus harness the variety of perspectives within a field of practice, rather than just introduce new philosophical perspectives and show how they can better serve the ends of practitioners. This underlines the importance, for applied philosophers adopting this insider‐outsider perspective, of being alert to the heterogeneity of social practices and institutions. And it suggests that part of the distinctive contribution of philosophers is to mediate between the ways in which such practices and institutions are currently conceived—and by whom—and the ways in which they could be reconceived.

## 5 BOTH TRUTH AND CONSEQUENCES

We have argued that applied philosophy sits between more abstract, philosophical scholarship, on the one hand, and practice, on the other, in so far as it reflects both a concern with truth‐seeking and a concern with the pragmatic ends of practitioners and fields of practice. We now turn our attention to the supposed methodological costs of applied philosophy. We suggest that a concern for particular outcomes and, in particular, a concern for the consequences of doing philosophy can itself be part of good philosophical method. Indeed, the insider‐outside position occupied by applied philosophers makes it especially necessary for them to consider the consequences of their philosophising.

We begin with some reflection on what philosophy is for. Our discussion so far has highlighted a tension between a more dispassionate view of the function of philosophy as the unconstrained pursuit of truth or the development of abstract, rational argument, and a more pragmatic view, which sees philosophy as, at least sometimes, answerable to the interests and commitments of non‐philosophers. The worry we started from is that the latter involves a concern for and attention to the consequences of philosophising that undermine its methods. This, however, expresses a fairly restricted view of the role and significance of philosophical practice. In her 1941 text “The Language of Political Theory,” Margaret MacDonald develops a more expansive view, arguing that the fact that philosophical arguments and claims can have profound effects is philosophically relevant. Despite their not saying anything that can be straightforwardly empirically verified or falsified, philosophical theories and statements have practical and psychological effects. Not to recognise this, MacDonald argues, is bad philosophy.

Philosophical theories and claims, MacDonald suggests, picture the world as being a certain way—emphasising the importance of certain constructs and relationships, and downplaying others. Sometimes one such theoretical framing will better account for the set of salient facts, or draw on a more credible set of facts, compared to alternative theories. But two philosophical theories can (in principle) both take account of exactly the same set of facts, frame them very differently, and make divergent and even contrary interpretive claims in relation to them. If a philosophical theory captures people's imagination and appears salient to them, they can use it to interpret and shape the world around them. Failure or refusal to recognise these effects, MacDonald argues, misses the “point” of philosophy—that is, why it is meaningful to engage in this kind of creative redescription of the world and why philosophers do it. While many philosophical arguments are not offered in response to practical problems, arguments in ethics and political philosophy typically *are*. If the impact of philosophising on practical issues is cast as irrelevant or outside the scope of philosophy, it becomes very difficult to understand what it is that philosophers are supposed to be doing—what it is that philosophy is *for*. Moreover, it fails to make sense of the kind of claims that philosophers make—claims that are not factual but linguistic, logical, ethical, and behavioural prescriptions on the basis of interpretations of fact.

A concern that might arise here is that this account gives applied philosophy *consequences* but denies it *truth*. MacDonald criticises “general, metaphysical theories” of political philosophy that “seek to reduce all political obligation to the application of an almost magical formula” (1941, 111). Instead, she notes: “The value of the political theorists, however, is not in the general information they give about the basis of political obligation but in their skill in emphasizing at a critical moment a criterion which is tending to be overlooked or denied” (112). Philosophy that takes seriously its own consequences thus moves away from the provision of general claims, because different social and political circumstances will throw up different problems and considerations to which germane philosophical theories will respond. Lorna Finlayson, in a commentary on MacDonald's essay, interprets this as meaning that “statements of political theory were never in the business of capturing or failing to capture truths about the world” and that they are instead “practical interventions in response to practical situations” (Finlayson 2015).

We suggest that this choice between truth and practicality represents a false dichotomy. A concern with both truth and practical consequences will come together for applied philosophers seeking to meaningfully contribute to practical social and institutional problems. Philosophical analysis may be localised but not thereby be less “true”—rather, it answers particular, time‐and‐situation‐constrained questions about what we should do and why. Outside philosophy the realm of “truth” is not reserved only for timeless, abstract generalities; scientific, sociological, and historical truth is not always expected to be abstract or widely generalisable, and nor, we suggest, should philosophical truth. Indeed, truth‐seeking in any domain must attend to the scope of applicability and limitations of any truth claims. But, moreover, one of the reasons that philosophical truth is liable to be localised in practical settings is because of the consequences of philosophers recommending or counselling against particular courses of action. The ways that philosophical interventions into policy and practice are likely be interpreted are themselves an important part of the context which philosophers are responding to and to which their theories and judgements pertain. This is not to suggest that such consequences should be the only or primary concern of such philosophers but rather to suggest that a failure to take them into account risks forsaking the truth of philosophical advice.

A helpful analogy here is the idea of performativity in economics. In complex, human systems, models and predictions can become self‐fulfilling or self‐sabotaging if knowledge of them leads people to change their behaviour. Performativity describes situations where the existence and use of theories or models shapes the world to be *more* like that described in the model; counter‐performativity describes situations where the existence and use of models shapes the world to become *less* like it is theorised to be. These phenomena are familiar in economics, where models have ended up both rationalising markets by changing the way that traders made decisions (MacKenzie and Millo 2003) and destabilizing markets by measuring market volatility and so enabling bets to be made on future volatility (Wilson 2021). The truth of economic models depends on the interplay between the theory of the economy and the economy itself. And this means that the truth of these models is dependent on context‐specific facts about how they are in fact interpreted and deployed. The performativity of models and theories problematises a clean theory/world distinction, because theories and theorising don't sit outside the domains that they pronounce upon in any decisive sense. The truth of models and theories is, in principle, and at least sometimes in practice, dependent on their correctly characterising the world qua a world that includes the theory and theorising about it.

Applied philosophical theories and views similarly make claims, including prescriptive claims, about areas of practice from which they can't be seen as fully separate. While the consequences of applied philosophical theories may not typically be as spectacular and far reaching as those of economic models, they can nonetheless be relevant to the truth of the theories. An example from our own work will help to illustrate what this might look like in practice. Philosophers and ethicists working in health care and health policy settings are often asked to develop frameworks, checklists, or schematics that reflect a simplified or distilled version of their view. Such requests may come from journal editors and reviewers, or practitioner collaborators and colleagues, and typically reflect a well‐meaning attempt to ensure that research is in a form that is accessible to and usable by busy practitioners who don't have philosophical training. In our own health care ethics research we have, on the whole, resisted such calls, largely out of concern that checklists and frameworks can close down the open‐ended deliberation that we think ethical health care practice requires. We worry that such decision‐making tools risk implying that ethics can be exhausted by following a predetermined sequence of steps or by considering a determinate list of values. Instead, we tend to emphasise the need for ongoing critical reflection on health care practice, and where we do provide guiding or framing questions, we emphasise that they are provisional and not comprehensive. While this approach resonates with some health care professionals, our work is, as a result of our schema avoidance, undoubtedly less “impactful” than it might otherwise be and is regularly rejected by medical and health services research journals on account of being insufficiently practical.

To capitulate and adopt a more schematic ethical approach might feel like a methodological compromise too far: the demands of health care practitioners for operational ethical frameworks would require us to lower or distort our philosophical standards in service of health care goals. But a “truth and consequences” conception of applied ethics suggests another possibility. If the practical and conceptual constraints of health care research and practice make it difficult or even impossible for many non‐philosophers to recognise the value of more deliberative and open‐ended ethics, and to apply it in their work, then the injunction that they *should* do so may often just be false. If, for example, the workload of many health care practitioners means that extended deliberative ethical practice would burden them with demanding and unachievable responsibilities, then philosophical injunctions to engage in this additional work may themselves be unethical. In other words, a lack of engagement with our philosophical research might not indicate a failure on the part of professionals to appreciate good philosophy when they see it but rather expose some of the shortcomings of our arguments in reflecting the ethical realities of health care practice. Ethical advice that does not meet its intended beneficiaries where they are will not have the potential to be meaningfully action guiding and so will not, in some key respects, reflect what the relevant moral actors should do.

The answer to this predicament need not, however, be for us to reluctantly supply reductive (and insincere) ethical frameworks in the hope that they will be better than nothing. The insider‐outsider stance that we have set out in this essay suggests that it is not helpful to conceive of a sharp binary between philosophical advice “hitting” or “missing” the intended audience. Rather, it highlights the potential to work expansively within the conventions and constraints of practice, for example by focusing on longer‐term goals, emphasising educative and bridge‐building strategies, and exploring promising routes towards less schematic ethical thinking within practice. In the short term there may be compromises that we can embrace, including recognising the value of more schematic decision‐making tools, while making efforts to ensure that these are appropriately caveated and contextualised. Another insider‐outsider strategy is to recognise and engage with the diversity that practitioners exhibit in their capacity and inclination to engage with more reflective ethical thinking. Working with people who are already predisposed to non‐schematic ways of thinking may indicate ways to communicate their enthusiasm to more sceptical colleagues.

We have painted a picture of applied philosophy as a set of scholarly activities that does not involve a choice between adopting the goals of practitioners or public policymakers, on the one hand, and adopting a goal of unconstrained truth‐seeking, on the other. Consideration of the consequences of philosophical practice can lead not away from philosophical truth but towards it. Applied philosophers can adopt an insider‐outsider stance, whereby their scholarly practice is constrained by the goals of a practice in a broad sense, without it being constrained by the goals of practitioners in a narrow sense. Understanding and recognising the consequences of philosophising in such contexts is absolutely central to its success. For a failure to do so may lead to a failure to in fact meaningfully support the goals of practice in the broad sense, leaving applied philosophy as a practice without a point. This calls for truth‐seeking within, rather than only outside, a specific domain of practice—where that domain also encompasses the practice of the applied philosopher.

## 6 METHOD AND MORALITY

We conclude, in this section, with some reflections on the methodological distinctiveness of applied philosophy vis‐à‐vis more theoretical philosophical approaches.

While the *content* of applied philosophy is different from that of more traditional, abstract philosophical work, it is less clear that there is a distinctive applied philosophical *method* (Beauchamp 1984). A method is, broadly speaking, a means of achieving some end. A scholarly method typically captures the activities that enable the pursuit of certain forms of knowledge—what you, as a scholar, must do in order to be justified in making knowledge claims in a particular domain. Methods can be distinguished from broader goals, on the one hand, and more specific practices by which methods are enacted, on the other. But it is worth noting that each of these distinctions is somewhat vague. A method can be understood as a general description of activities that is neither so broad as to capture only headline aims and ends nor so specific as to capture everything that actually goes on in particular laboratories, offices, and research settings.

While applied philosophers focus their research on particular concepts and topics, they undertake activities and analysis similar to those of their more theoretical counterparts. But although the practices of applied philosophers substantially overlap with those of theoretical philosophers, they also differ. Applied philosophers frequently read and engage with different material as a basis for their scholarly work—including qualitative and quantitative data, as well as academic scholarship from other disciplines—and present and publish their work in different media and forums, which at least sometimes involves tailoring their language, arguments, and topics for specific audiences.

The goals, too, of applied philosophers are different from those of at least some theoretical philosophers. This is because applied philosophers can be seen to be answerable in two directions—both towards their philosophical colleagues and philosophical standards of argumentative rigour, on the one hand, and towards policymakers, practitioners, and the publics they serve, on the other. As this essay has explored, the work of applied philosophers is, albeit in variable ways, responsive to practice. Responsiveness to practice amounts to more than mere restrictions on subject matter. Crucially, the unconstrained argument and critique that Brock identifies as central philosophical virtues are downplayed. It is not that applied philosophers are no longer engaged in truth‐seeking or knowledge‐seeking, nor that they are committed to ignoring ideal theory. Rather, there is a pragmatic quality to applied philosophical truth‐seeking—truth is not necessarily sought as an end in itself but sought as a means to support particular practices and forms of knowledge‐making.

Some applied philosophical work relatively uncontroversially involves distinct methods. Empirical bioethics is a well‐established field that uses empirical methods to help address normative questions (Ives et al. 2017). The empirical work performed by bioethicists leans on empirical methods from other disciplines—qualitative methods such as content analysis and thematic analysis, as well as quantitative methods such as descriptive statistics and inferential statistics on data collected from surveys and sources such as medical records (Wangmo and Provoost 2017). Distinctively philosophical methods may, however, be employed in the use and interpretation of empirical data to help answer normative questions. While there is limited agreement between bioethicists as to what method they use to integrate normative and empirical work (if they recognise themselves as using a method at all), methods identified include reflective equilibrium, reflective balancing, and dialogical empirical ethics (Wangmo and Provoost 2017). These focus on bottom‐up rather than top‐down reasoning—abductively reasoning from specifics to pro tanto generalisations, rather than deductively reasoning from generalisations to specific conclusions. Taking, at least to some extent, the values, concepts, and reasoning found in the world at face value and working critically with them to develop philosophical theories that seek primarily to explain or build on them, rather than explain them away, represents a distinctive approach to theoretical and conceptual analysis. This work is arguably also of crucial substantive importance in ethics because at least some portion of ethical uncertainty and disagreement is immanent in practices, such that there is every risk of not understanding what is at stake without a good measure of bottom‐up work.

We recognise that the extent to which these differences reflect a method distinct from scholarly philosophy is somewhat open. Applied philosophy encompasses a wide spectrum of activity, parts of which are more methodologically aligned with scholarly philosophy, and parts of which are methodologically distant. But, crucially, the differences in methods, goals, and practices across this spectrum are not best understood as potentially concerning deviations from a gold‐standard philosophical method. Instead, they can represent distinct philosophical approaches that are intentional, thoughtful, and highly attuned to the contexts in which they operate—and are no less philosophically virtuous or truth oriented as a result.

Applied philosophy has gained a good deal more visibility and institutional recognition since Brock's “Truth or Consequences” was published. Over this period, it has become more commonplace. Arguably the questions Brock raises have become ever more important: we need to consider the trade‐offs that arise when taking philosophy out of the seminar room and into the field. We have suggested, however, that it is not helpful to think of these in dichotomous ways—choosing either scholarly or civic virtues. Rather, scholarship can encompass a wide variety of engagements and commitments, and scholarly virtues need to adapt and expand accordingly. More specifically, we have argued that there is no reason to locate a concern for truth (or for consequences) on one side of a scholarship/practice distinction. If our arguments are correct, this will introduce methodological and moral challenges and various balancing acts into the domain of the applied philosopher, including the management of power dynamics and the resolution of conceptual, strategic, and practical disagreements (Scully 2019). But the high‐stakes kinds of dilemma with which this paper began can be seen as but one feature of a pervasive set of ethical judgement calls demanded by applied philosophy. In the vast majority of instances, these judgements can be reframed as being about scholarly choices rather than choices between scholarship and non‐scholarly practice. This is something that is already familiar from other fields, such as applied and social sciences. Indeed, philosophers interested in the methodological and moral challenges of fieldwork might do well to begin by considering the substantial literature that exists in other areas on themes such as insider‐outsider research, translational scholarship, and embedded fieldwork (Bruskin 2019; Engebretsen et al. 2020; Milligan 2014; Vindrola‐Padros et al. 2017).

The goals and interests of policymakers, practitioners, and the public are not necessarily served by the arguments and reasoning developed in the most theoretical depths of philosophy; applied philosophers actively and intentionally seek to bridge this gap. We are optimistic about the possibility of sustained collaboration, mutual support, and genuinely collective learning involving applied philosophers. This optimism stems in part from seeing philosophy, particularly applied philosophy, as a practice with a point—a practice that seeks to have effects outside its own domain—and so as a practice that can gain from listening to and speaking to diverse practitioners, including non‐philosophers, and paying attention to how ideas are received and acted upon. Although there are good reasons for applied philosophers to sustain strong relationships with the philosophy academy and to practice the virtues it supports, there is no reason to think that philosophers should carefully limit the time they spend working in applied settings. On the contrary, nurturing the range of capabilities and virtues that are inherent in applied philosophy depends on paying dedicated attention to, and practising, them. Deliberate engagement with particular public and professional domains and long‐term collaboration with practitioners has the potential to enrich and improve applied philosophical scholarship, rather than to dilute or threaten it.

## Footnotes

^1^
In the forty years after Brock joined the President's Commission, he became one of the most influential and widely respected figures in bioethics. “Truth or Consequences” was a product of his first foray into the “sharp end” of applied philosophy, written at a time when the experience of moving outside his host philosophy department (at Brown University) was relatively novel. Since then, the possibilities for philosophers working “in the field” have expanded significantly—a development heralded and furthered by the work of Brock and others—and it is this space of possibility that concerns us here.

^2^
We do not mean to suggest that critical reflection, deployed in a “cool hour,” is more likely to lead to moral and philosophical insight or truth than quicker, responsive thinking that is done in practical decision‐making contexts (cf. Williams's [1982] critique of Sidgwick's strategy of divorcing moral theory from moral practice in *The Methods of Ethics*). Rather, we want to suggest that applied philosophers must manage and balance a number of different concerns and ends, some of which are more proximate and related to practice as currently conceived and applied, and some of which are more distant and related to practice expansively envisioned and reconceived. Neither of these perspectives is prior to or more authentically philosophical than the other. Indeed, the distinctive contribution of the applied philosopher partly consists in her ability to move between these frames and to occupy the spectrum they bookend.

## References

[meta12644-bib-0001] Archard, David . 2021. “Philosophical Advice.” Philosophy 96: 603–23.

[meta12644-bib-0002] Arras, John D. 2007. “The Way We Reason Now: Reflective Equilibrium in Bioethics.” In The Oxford Handbook of Bioethics, edited by Bonnie Steinbock , 46–71. Oxford: Oxford University Press.

[meta12644-bib-0003] Beauchamp, Tom L. 2005. “The Nature of Applied Ethics.” In A Companion to Applied Ethics, edited by R. G. Frey and Christopher Heath Wellman , 1–16. Oxford: Blackwell.

[meta12644-bib-0004] Beauchamp, Tom L. 1984. “On Eliminating the Distinction Between Applied Ethics and Ethical Theory.” Monist 67, no. 4: 514–31.

[meta12644-bib-0005] Beauchamp, Tom L. , and James F. Childress . 2019. Principles of Biomedical Ethics. 8th edition. New York: Oxford University Press.

[meta12644-bib-0006] Broadbent, Alex . 2019. Philosophy of Medicine. New York: Oxford University Press.

[meta12644-bib-0007] Brock, Dan W. 1987. “Truth or Consequences: The Role of Philosophers in Policymaking.” Ethics 97, no. 4: 786–91. 11658836 10.1086/292891

[meta12644-bib-0008] Bruskin, Signe . 2019. “Insider or Outsider? Exploring the Fluidity of the Roles Through Social Identity Theory.” Journal of Organizational Ethnography 8, no. 2: 159–70.

[meta12644-bib-0009] Caplan, Arthur L. 1980. “Ethical Engineers Need Not Apply: The State of Applied Ethics Today.” Science, Technology, and Human Values 6, no. 33: 24–32. 10.1177/016224398000500403 11665127

[meta12644-bib-0010] Carel, Havi . 2016. Phenomenology of Illness. 1st edition. Oxford: Oxford University Press.

[meta12644-bib-0011] Cartwright, Nancy . 1999. The Dappled World: A Study of the Boundaries of Science. Cambridge: Cambridge University Press.

[meta12644-bib-0012] Charon, Rita . 2006. Narrative Medicine: Honoring the Stories of Illness. Oxford: Oxford University Press.

[meta12644-bib-0013] Daniels, Norman . 1996. Justice and Justification: Reflective Equilibrium in Theory and Practice. Cambridge: Cambridge University Press.

[meta12644-bib-0014] Dupré, John . 1993. The Disorder of Things: Metaphysical Foundations of the Disunity of Science. Cambridge, Mass.: Harvard University Press.

[meta12644-bib-0015] Dzur, Albert W. , and Danielma Lessard Levin . 2004. “The ‘Nation's Conscience’: Assessing Bioethics Commissions as Public Forums.” Kennedy Institute of Ethics Journal 14, no. 4: 333–60. 15812983 10.1353/ken.2004.0042

[meta12644-bib-0016] Engebretsen, Eivind , et al. 2020. “Towards a Translational Medical Humanities: Introducing the Cultural Crossings of Care.” Medical Humanities 46, no. 2: e2. 32341131 10.1136/medhum-2019-011751 PMC7402465

[meta12644-bib-0017] Farmer, Paul , and Nicole Gastineau Campos . 2004. “Rethinking Medical Ethics: A View from Below.” Developing World Bioethics 4, no. 1: 17–41. 15086372 10.1111/j.1471-8731.2004.00065.x

[meta12644-bib-0018] Finlayson, Lorna . 2015. “‘Never Go to Sleep’: Political Theory as a Foreign Language.” Methods in Ethics: The Virtual Issue of the Aristotelian Society 3, edited by Ben Colburn , 126–34. London: Aristotelian Society.

[meta12644-bib-0019] Flanagan, Owen . 1991. Varieties of Moral Personality: Ethics and Psychological Realism. Cambridge, Mass.: Harvard University Press.

[meta12644-bib-0020] Giubilini, Alberto , and Francesca Minerva . 2012. “An Open Letter from Giubilini and Minerva.” Journal of Medical Ethics Blog. March 12. https://blogs.bmj.com/medical‐ethics/2012/03/02/an‐open‐letter‐from‐giubilini‐and‐minerva/.

[meta12644-bib-0021] Giubilini, Alberto , and Francesca Minerva . 2013. “After‐Birth Abortion: Why Should the Baby Live?” Journal of Medical Ethics 39: 261–63. 22361296 10.1136/medethics-2011-100411

[meta12644-bib-0022] Glackin, Shane N. 2019. “Grounded Disease: Constructing the Social from the Biological in Medicine.” Philosophical Quarterly 69, no. 275: 258–76.

[meta12644-bib-0023] Ives, Jonathan , et al. 2017. Empirical Bioethics: Theoretical and Practical Perspectives. Cambridge: Cambridge University Press.

[meta12644-bib-0024] Kamm, Frances M. 1990. “The Philosopher as Insider and Outsider.” Journal of Medicine and Philosophy 15, no. 4: 7–20. 10.1093/jmp/15.4.347 2230618

[meta12644-bib-0025] Kitcher, Philip . 1996. The Lives to Come: The Genetic Revolution and Human Possibilities. New York: Simon and Schuster.

[meta12644-bib-0026] MacDonald, Margaret . 1941. “The Language of Political Theory.” Proceedings of the Aristotelian Society 41: 91–112.

[meta12644-bib-0027] MacKenzie, Donald , and Yuval Millo . 2003. “Constructing a Market, Performing Theory: The Historical Sociology of a Financial Derivatives Exchange.” American Journal of Sociology 109, no. 1: 107–45.

[meta12644-bib-0028] Milligan, Lizzi . 2014. “Insider‐Outsider‐Inbetweener? Researcher Positioning, Participative Methods and Cross‐Cultural Educational Research.” Compare: A Journal of Comparative and International Education 46, no. 2: 235–50.

[meta12644-bib-0029] Nordenfelt, Lennart . 1995. *On the Nature of Health: An Action‐Theoretic Approach* . 2nd rev. and enl. ed. Dordrecht: Kluwer.

[meta12644-bib-0030] Scully, Jackie Leach . 2017. “Feminist Empirical Bioethics.” In Empirical Bioethics: Theoretical and Practical Perspectives, edited by Jonathan Ives et al., 195–221. Cambridge: Cambridge University Press.

[meta12644-bib-0031] Scully, Jackie Leach . 2019. “The Responsibilities of the Engaged Bioethicist: Scholar, Advocate, Activist.” Bioethics 33, no. 8: 872–80. 31532850 10.1111/bioe.12659

[meta12644-bib-0032] Valles, Sean A. 2018. Philosophy of Population Health Science: Philosophy for a New Public Health Era. London: Routledge.

[meta12644-bib-0033] Venkatapuram, Sridhar . 2011. Health Justice: An Argument from the Capabilities Approach. Cambridge: Polity.

[meta12644-bib-0034] Vindrola‐Padros, Cecilia , et al. 2017. “The Role of Embedded Research in Quality Improvement: A Narrative Review.” BMJ Quality and Safety 26, no. 1: 70–80. 10.1136/bmjqs-2015-004877 PMC5256405 27129492

[meta12644-bib-0035] Walker, Margaret Urban . 2007. Moral Understandings: A Feminist Study in Ethics. 2nd edition. New York: Oxford University Press.

[meta12644-bib-0036] Wangmo, Tenzin , and Veerle Provoost . 2017. “The Use of Empirical Research in Bioethics: A Survey of Researchers in Twelve European Countries.” BMC Medical Ethics 18: 79. 29273030 10.1186/s12910-017-0239-0 PMC5741864

[meta12644-bib-0037] Williams, Bernard . 1982. “The Point of View of the Universe: Sidgwick and the Ambitions of Ethics.” Cambridge Review 7: 183–91.

[meta12644-bib-0038] Wilson, James . 2021. Philosophy for Public Health and Public Policy: Beond the Neglectful State. Oxford: Oxford University Press.

[meta12644-bib-0039] Wilson, James . 2009. “Towards a Normative Framework for Public Health Ethics and Policy.” Public Health Ethics 2, no. 2: 184–94.

[meta12644-bib-0040] Wolff, Jonathan . 2011. Ethics and Public Policy: A Philosophical Inquiry. New York: Routledge.

[meta12644-bib-0041] Wolff, Jonathan . 2019. Ethics and Public Policy: A Philosophical Inquiry. 2nd ed. London: Routledge.

